# Protected areas alleviate climate change effects on northern bird species of conservation concern

**DOI:** 10.1002/ece3.1162

**Published:** 2014-07-03

**Authors:** Raimo Virkkala, Juha Pöyry, Risto K Heikkinen, Aleksi Lehikoinen, Jari Valkama

**Affiliations:** 1 Natural Environment Centre, Finnish Environment InstituteMechelininkatu 34 a, P.O. Box 140, FI-00251, Helsinki, Finland; 2 Finnish Museum of Natural HistoryP.O. Box 17, FI-00014, University of Helsinki, Finland

**Keywords:** Bird species richness, climate change, habitat, protected areas, resilience, species of conservation concern

## Abstract

Global climate change is a major threat to biodiversity, posing increasing pressures on species to adapt in situ or shift their ranges. A protected area network is one of the main instruments to alleviate the negative impacts of climate change. Importantly, protected area networks might be expected to enhance the resilience of regional populations of species of conservation concern, resulting in slower species loss in landscapes with a significant amount of protected habitat compared to unprotected landscapes. Based on national bird atlases compiled in 1974–1989 and 2006–2010, this study examines the recent range shifts in 90 forest, mire, marshland, and Arctic mountain heath bird species of conservation concern in Finland, as well as the changes in their species richness in protected versus unprotected areas. The trends emerging from the atlas data comparisons were also related to the earlier study dealing with predictions of distributional changes for these species for the time slice of 2051–2080, developed using bioclimatic envelope models (BEMs). Our results suggest that the observed changes in bird distributions are in the same direction as the BEM-based predictions, resulting in a decrease in species richness of mire and Arctic mountain heath species and an increase in marshland species. The patterns of changes in species richness between the two time slices are in general parallel in protected and unprotected areas. However, importantly, protected areas maintained a higher level of species richness than unprotected areas. This finding provides support for the significance and resilience provision of protected area networks in preserving species of conservation concern under climate change.

## Introduction

Global climate change is a major threat to biodiversity (Pereira et al. 2010), already affecting species populations and communities (Hickling et al. 2006; Parmesan 2006; Chen et al. 2011) and is projected to cause accelerating poleward and upward range shifts in different taxa (Araújo et al. 2011; Barbet-Massin et al. 2012). The potential impacts of climate change on species distributions has predominantly been assessed with bioclimatic envelope models (BEMs), or ecological niche models (ENMs), whereby the relationships between present-day distributions and climatic variables are modeled and then used to forecast the changes in a suitable climate space for species (Pearson and Dawson 2003; Thuiller et al. 2005; Heikkinen et al. 2006a; Virkkala et al. 2010; Araújo and Peterson 2012; Barbet-Massin et al. 2012). BEMs have certain limitations (Heikkinen et al. 2006a; Sinclair et al. 2010; Sieck et al. 2011) but when applied with caution, they can provide useful broad-scale projections of the direction and magnitude of potential changes in species distributions (Araújo and Peterson 2012). These projections may consequently be used as a basis for conservation planning assessments, to examine the potential species losses, turnover and gain in conservation areas, and future gaps in the protected area (PA) network (Hannah et al. 2007; Hole et al. 2009, 2011; Araújo et al. 2011).

One of the limitations of BEMs is validation; these models are typically employed to provide forecasts for future changes which have not yet happened. A proper validation of the models would require temporally independent datasets. Unfortunately, a lack of distributional data from two time periods often hampers such tests. The few studies available have shown good to fair predictive performance for BEMs (Araújo et al. 2005; Hijmans and Graham 2006; Kharouba et al. 2009; Eskildsen et al. 2013), but model performance varies considerably between species and species groups. What appears to be lacking even more are studies that integrate projections of species range shifts and actual observed changes in distributions, with the geographic variation of the present-day PAs. This is a shortcoming because the PA network is one of the main instruments that can help species adapt to climate change (Coetzee et al. 2009; Hole et al. 2009; Araújo et al. 2011). One recent study showed that protected areas may facilitate species range expansions and individual movement in a highly fragmented landscape (Thomas et al. 2012). This suggests that the shifts in species distributions, projected by, for example, BEMs, may be realized more readily in landscapes with suitable protected habitats for the species than in human-influenced landscapes with little protected habitat. In addition, species loss may occur more slowly in areas with larger amounts of protected habitat. Thus, the spatial distribution of a PA network may affect how well the BEM-based forecasts are realized, but to what extent is so far poorly known.

Virkkala et al. (2013a,b) projected distributional changes for 100 bird species of conservation concern inhabiting forest, mire, marshland, and Arctic mountain habitats by using BEMs and climate scenario data for the years 2051–2080 in Finland, northern Europe. Moreover, they related the projected changes in climatic suitability to the amount of protected preferred habitat of the study species in each 10-km^2^ in Finland. The climatically suitable areas were generally predicted to shift northwards, but overall the probability of occurrence of species in all habitat types (except marshland birds) was projected to decrease (Virkkala et al. 2013a). This predicted decline was greater in unprotected than in protected areas for species of forests, mires, and Arctic mountains. In addition, in species of mires, marshlands, and Arctic mountains, a high proportion of protected habitat (35–95%) was included in the most suitable squares (the highest 5% of suitability squares) in the scenarios in 2051–2080, suggesting that protected areas can cover a high proportion of the future occurrences of bird species (Virkkala et al. 2013b). In contrast, for forest birds in the southern and central parts of Finland, the efficiency of the PA network was projected to be insufficient.

Here, we will study whether recent changes in bird species ranges in Finland show emerging trends that are in the same direction as the longer-term predictions of distributional changes described by Virkkala et al. (2013a,b). The reasoning for such a comparison is that in our study area a notable summertime warming has already occurred in the last ca. 20 years (Heino et al. 2008; Tietäväinen et al. 2010), which has apparently triggered the first distributional changes in birds (Brommer et al. 2012) and also in other species groups such as butterflies (Pöyry et al. 2009). Our study focuses on the same set of species that Virkkala et al. (2013a,b) studied. The BEMs developed there are compared with changes in bird species distributions extracted from the bird atlases for which data were collected in two time periods, 1974–1989 and 2006–2010. We relate the observed distributional changes to the PA network across three latitudinal zones, with the four focal habitats analyzed separately, and thereby address the following questions: (1) Are the observed distributional changes in the same direction as those of predicted longer-term range shifts of species? (2) Are the distributional changes of species more pronounced in unprotected than in protected areas, that is can a protected area network be resilient in relation to climate change in preserving species of conservation concern, and (3) do species with a northern versus southern distributional pattern differ?

## Materials and Methods

### Bird atlases

We used data from three bird atlas studies carried out in Finland: field work was carried out in 1974–1979, 1986–1989 and in 2006–2010 (Hyytiä et al. 1983; Väisänen et al. 1998; Valkama et al. 2011; Brommer et al. 2012). We pooled the information of the first two bird atlas surveys carried out in 1974–1979 and in 1986–1989 (Väisänen et al. 1998). This was done because the third atlas in 2006–2010 was much more thorough (for categories of survey activity, see Väisänen 1989) than the first two (see Valkama et al. 2011; Brommer et al. 2012), and atlas studies are susceptible to variations in survey effort (see Kujala et al. 2013). Importantly, we also wanted to ensure the methodological comparability with the earlier bioclimatic envelope modeling studies of Virkkala et al. (2013a,b), who made predictions on bird species of conservation concern and used European bird atlas data compiled mostly between 1971 and 1995 (see Hagemeijer and Blair 1997). Surveys for the Finnish atlases were carried out using a uniform grid system of 10 × 10 km, and the level of breeding status of bird species (recorded by bird observers) and survey activity (calculated based on number of species observations with varying breeding status included, Väisänen et al. 1998) in each square was recorded.

The breeding status of bird species recorded in each of the grid squares was assessed using four classes: 0 = not found, 1 = breeding possible (e.g., singing or displaying male observed once in a typical nesting habitat), 2 = breeding probable (e.g., singing or displaying male with a persistent territory observed, or female or pair present on more than one day in the same place, or bird observed building a nest), 3 = confirmed breeding (Väisänen 1989). For the analyses of this study, we combined classes 1, 2, and 3 to indicate species presence.

The atlas surveys graded the survey activity in each square according to six categories: 0 = no observations, 1 = occasional observations, 2 = fair surveys, 3 = satisfactory survey of the square, 4 = well surveyed and 5 = thoroughly surveyed squares (Väisänen et al. 1998).

To control for the potential impacts of variation in survey efficiency, we only included squares with at least fair surveys (2–5) in both periods (1974–1989 and 2006–2010) and with the maximum difference of two categories of survey efficiency between the two periods. Originally, there were 3813 grid squares covering the entire country, of which 3399 were included in our analyses based on these survey effort requirements. In addition, we used survey effort as a covariable in all subsequent analyses.

### Bird species

We focused on the same bird species that were included in the study by Virkkala et al. (2013a,b), who forecasted the future range shifts of 100 bird species of conservation concern using BEMs. However, we excluded seven species which were not observed in the atlases but are expected to expand their ranges to Finland by 2051–2080, and three breeding bird species which showed a clear overall difference in survey effort between the two atlases: the Eurasian pygmy owl *Glaucidium passerinum*, the white-backed woodpecker *Dendrocopos leucotos* and the Arctic redpoll *Carduelis hornemanni* (see Table S1, and Virkkala et al. 1993; Saurola 2008; Lehikoinen et al. 2011; Valkama et al. 2011). Thus, we considered a total of 90 land bird species of conservation concern which were all included in Virkkala et al. (2013a,b).

The 90 studied species were selected using a number of classifications of conservation concern and the critical categories in them (see Virkkala et al. 2013a,b): the European Union's Birds Directive species (Annex I), species of European conservation concern (SPEC1–SPEC3) (BirdLife International 2004a), species of Arctic or boreal biomes for important bird areas in Europe (IBA) (Heath and Evans 2000), threatened species in the European Union (unfavorable conservation status) (BirdLife International 2004b), species of special responsibility in Finland (Rassi et al. 2001), red-listed species in Finland in 2010 (near-threatened and threatened species) (Rassi et al. 2010), and species preferring old-growth or mature forests in Finland (Virkkala et al. 1994; Väisänen et al. 1998; Virkkala and Rajasärkkä 2007). Our study species belonged to at least one of these classifications. We focused on the species from four main terrestrial habitats: forests, open mires, other wetlands (here regarded as marshlands), and mountain habitats.

Each bird species was related to its main habitat in Finland (Table S1). Of our 90 land bird species, 44 were classified as species of forests, 21 species of mires, 15 species of marshlands, and 10 species of Arctic mountain habitats (Table S1, see also Virkkala et al. 2013a,b). Of the species of mountain habitats, nine were regarded as species of mountain heaths and one as a species (bluethroat, *Luscinia svecica*) of mountain birch *Betula pubescens czerepanovii* woodlands.

In addition to habitat preferences, we divided bird species by their distribution pattern as a southern or northern species or as a species distributed over the whole country according to Virkkala and Rajasärkkä (2011a). All Arctic mountain species were northern, all except one mire species were northern, and most marshland species (12 out of 15 species) were southern (Table S1). Of the 44 forest species, 18 were southern, 13 were northern and six were distributed over the whole country.

### Habitat classification and protection of habitats

**Figure 1:**
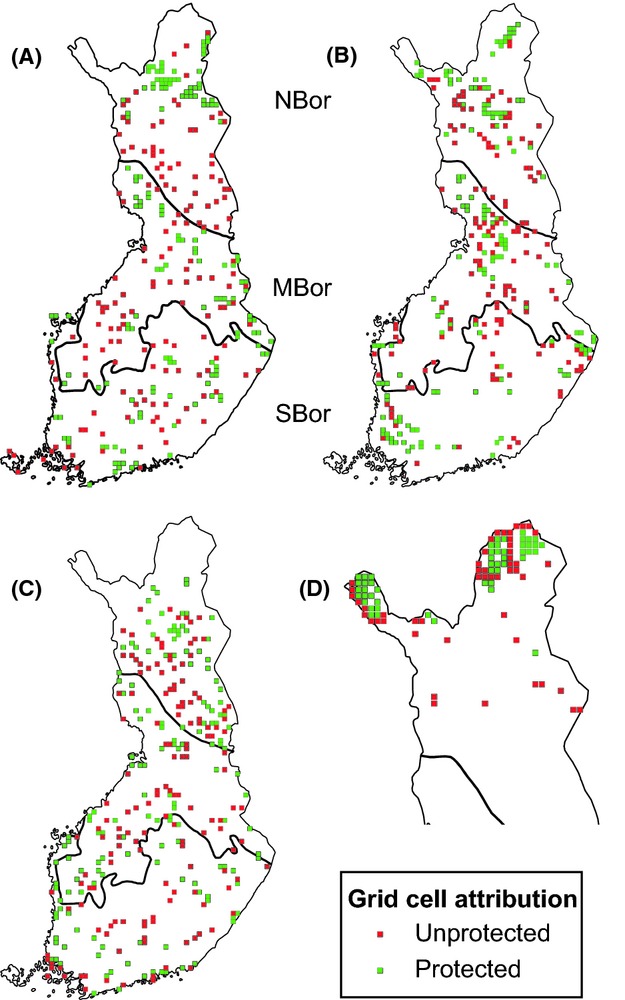
Location of protected and unprotected squares in the different vegetation zones. (A) forests, (B) open mires, (C) marshlands, (D) Arctic mountain heaths. SBor = southern boreal, MBor = middle boreal, NBor = northern boreal.

Biogeographically, Finland stretches through the boreal coniferous vegetation zone. Habitats and protected areas were therefore investigated separately in three main vegetation zones occurring in the country: the southern boreal, the middle boreal, and the northern boreal zones (see Fig. 1). The extent of the hemiboreal zone in the southwestern coast of Finland is small, and therefore it was combined with the southern boreal zone. In the northern boreal zone, mountain birch forms both the northernmost forests and the tree line.

Following Virkkala et al. (2013a,b), we employed CORINE land cover data (for detailed presentation, see Appendix S1) due to its complete spatial coverage of Finland (Härmä et al. 2004), and because of the homogeneity of the methodology used for the land cover classification at the pan-European level (EU countries).

The PA network consisted of strictly protected areas in which the economic use of habitats, such as logging or drainage of wetlands, is not allowed. (see Appendix S1). Protected areas belong to the IUCN categories Ia (Strict nature reserve), Ib (Wilderness area), II (National park) or IV (Habitat/species management area) (Dudley 2008). In contrast, land use patterns in unprotected areas include a number of important human influences, with intensive forest management for forestry purposes being the primary land use in forested habitats.

The amount of protected habitat differs considerably in Finland both from south to north and between habitat types (Table 1, Appendix S1, Fig. S1).

**Table 1:** Mean area of focal habitat protected, mean total area focal habitat (km^2^), and mean percentage of habitat protected in the 50 protected and the 50 randomly selected, unprotected squares of each habitat type in the different vegetation zones.

Habitat type	Southern boreal			Middle boreal			Northern boreal		
	Protected (km^2^)	Total (km^2^)	Protected (%)	Protected	Total (km^2^)	Protected (%)	Protected (km^2^)	Total (km^2^)	Protected (%)
Forest									
Protected squares	10.40	49.98	20.8	19.06	56.20	33.9	63.88	68.00	93.9
Unprotected squares	0.52	46.42	1.1	1.19	57.71	2.1	8.81	63.00	14.0
Open mire									
Protected squares	5.43	9.42	57.6	23.98	38.45	62.4	36.72	41.34	88.8
Unprotected squares	0.28	3.90	7.2	1.86	17.37	10.7	7.12	33.85	21.0
Marshland									
Protected squares	1.92	2.78	69.3	0.79	1.13	69.9	0.17	0.35	47.9
Unprotected squares	0.11	1.51	7.5	0.02	0.55	3.1	0.00	0.24	0.8
Mountain heath									
Protected squares	–	–	–	–	–	–	67.27	69.25	97.1
Unprotected squares	–	–	–	–	–	–	10.58	21.45	49.3

### Selection of the focal grid squares

For our comparisons, we selected 50 of the top 10 × 10 km squares representing the highest amount of protected habitat type (hereafter “protected” squares), measured separately for each vegetation zone and each habitat type. Thus, we selected 150 protected grid squares for forests, mires, and marshlands spread across the three vegetation zones (50 squares in each), and 50 grid squares for the mountain heaths in the northern boreal zone, totaling 500 focal protected grid squares (Fig. 1, Appendix S1).

In order to investigate the impact of protected areas on the distributional changes of bird species, we selected sets of 50 “unprotected” squares (10-km grid squares with only few or no protected areas) for each habitat type and vegetation zone as was done for the protected grid squares above. These focal 500 unprotected grid squares were then used for comparison in the subsequent analyses (Fig. 1). They were selected otherwise randomly in each zone but using a criterion that the lowest amount of protected focal habitat in the 50 protected squares was regarded as the minimum amount of habitat for the unprotected squares to be selected. This was done to ensure that there was also a focal habitat available in the unprotected squares. Because over 80% of the Arctic mountain heaths were situated in protected areas, unprotected squares for this habitat type were selected in order to have the lowest amount of protected mountain heath habitat (see Table 1).

### Climate data

We investigated the recent (1961–2012) variation and trends of three climate variables known to be among the main climatic drivers affecting bird species distributions (Heikkinen et al. 2006b; Huntley et al. 2007; Virkkala et al. 2010): mean temperature of April–June (*T*_AMJ_), annual temperature sum above 5°C (growing degree days, GDD5) and mean annual temperature (*T*_Ann_). These data of climate variables are based on 10 × 10 km gridded data obtained from the Finnish Meteorological Institute (Tietäväinen et al. 2010).

### Statistical analyses

We performed two main types of statistical analyses with the data. All analyses were performed within the R statistical environment, version 3.0.2. (R Development Core Team 2013).

In the first set of analyses we modeled as response variables, the overall changes in species richness of all species and three groups of bird species classified based on their distributional pattern (southern species, northern species, and species occurring in the whole country) between the atlas periods (1974–1989 and 2006–2010) and across the three vegetation zones. This was done to take into account the distribution patterns of species with all the data included in the analysis. For example, species richness of southern and northern species might have had different patterns (see Virkkala and Rajasärkkä 2011a,b; Brommer et al. 2012). Here, we included data from all the 3399 10-km grid squares. For response variables with normal error structure (all species and species occurring in the whole country), we employed linear mixed-effect models as implemented in the nlme library (function lme). For response variables with Poisson error structure (southern and northern species), we employed generalized linear mixed-effect models as implemented in the lme4 library (function glmer). The study period and vegetation zone (treated as ordered factors) and their interaction terms were included as fixed factors and the grid square as a random factor in the models. Survey effort was included as a fixed cofactor in both the atlas periods. As atlas datasets often show high levels of spatial autocorrelation between grid squares situated geographically closely to each other (e.g., Legendre 1993; Dormann 2007), we assessed the potential occurrence of spatial autocorrelation in our data by using the ncf library. As this atlas data was recorded by uniform grid squares, we included spatial autocovariate (ACV) in all four models (Augustin et al. 1996; Dormann et al. 2007). The ACV was calculated by using the spdep library as the average of the observed species richness values of the direct neighbors for each grid square.

In the second set of analyses, we focused on the observed changes in species richness between the atlas study periods (1974–1989 and 2006–2010) with a particular interest in the impact of protected areas. Thus, we included in these analyses the sets of protected and unprotected grid squares selected for each habitat type (Fig. 1). Mixed-effect models were fitted separately for forest, open mire, marshland, and Arctic mountain heath species richness as response variables. Again, for response variables with normal error structure (forest, mire, and Arctic mountain heath species) we employed linear mixed-effect models as implemented in the nlme library (function lme), and for response variables with Poisson error structure (marshland species) we employed generalized linear mixed-effect models as implemented in the lme4 library (function glmer). The study period, vegetation zone (treated as ordered factors), and the protection status of the grid square as well as interactions between all three terms were included as fixed factors and the grid square as a random factor. Survey effort was also included as a fixed cofactor in this analysis. A total of 300 grid squares (150 protected, 150 unprotected) were included in the models for forest, mire, and marshland species and 100 grid squares (50 protected, 50 unprotected) in the model for Arctic mountain heath species. Grid squares for species in different habitats were predominantly geographically more separated than in the first set of analyses, and thus we did not include spatial autocovariate in these models.

In both types of analyses, significances of model terms were calculated using an *F*-test for the linear mixed-effect models. In contrast, for the generalized linear mixed-effect models, significances were estimated using Wald's Type II χ^2^ test as implemented in the libraries pbkrtest and car. This was done because for the generalized models there are only iterative methods for calculating the denominator degrees of freedom for the included model terms (e.g., Venables and Ripley 2002; Baayen et al. 2008).

As a third analysis aiming to answer the first study question, we compared the observed changes in species richness (from 1974–1989 to 2006–2010) with the long-term changes in mean probability of occurrence based on bioclimatic envelope modeling (from 1971–1990 to 2051–2080, see Virkkala et al. 2013a) of each species group. Proportional decrease and increase are not statistically strictly comparable as, for example, a doubling increase from a probability of occurrence of 50% gives a value of 100%, but a similar decrease to half of that yields 25%. To avoid this discrepancy in statistical tests, we used a logarithmic ratio (log ratio) of percentage changes, where, for example, 100% (doubling) increase would be log (100/50) = +0.301 and 50% decrease (decrease to half) would be log (25/50) = –0.301.

## Results

**Figure 2:**
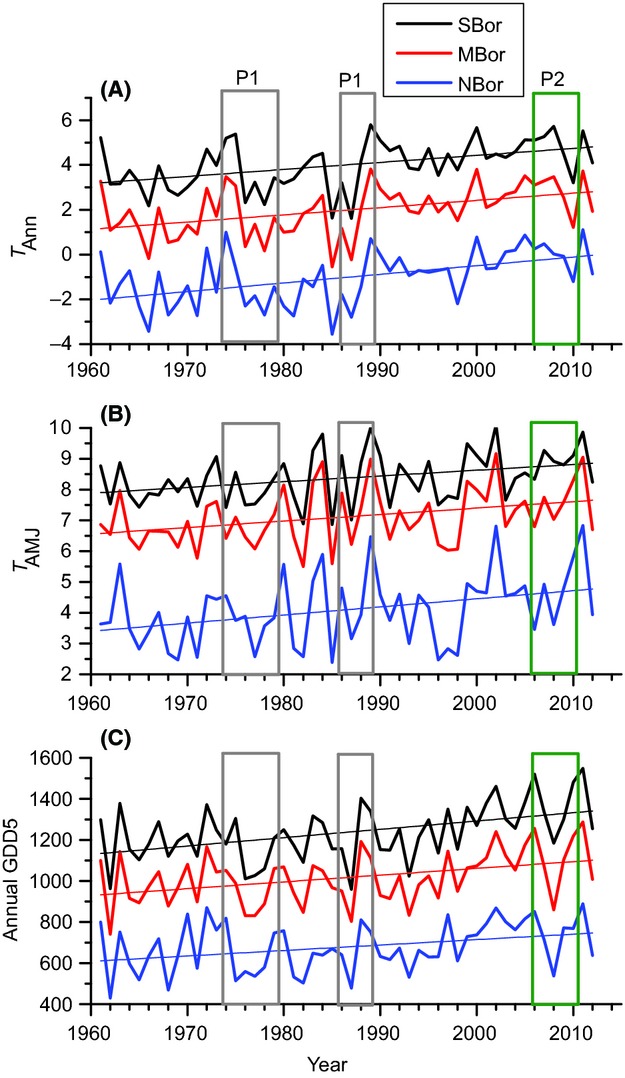
Mean annual temperature (A), April–June mean temperature (B), and annual temperature sum above 5°C (growing degree days) (C) in southern boreal (SBor), middle boreal (MBor), and northern boreal (NBor) zone between 1960–2012 with atlas periods shown in columns (P1–P3). Straight lines represent linear regressions fitted for each time series.

The mean April–June temperature (*T*_AMJ_) has risen by about 1°C and the mean annual temperature (*T*_Ann_) by about 2°C between the years 1961–2012 in Finland, with the highest annual temperature increase in the northern boreal zone (Fig. 2). On the other hand, the annual sum of growing degree days (GDD5) has increased most in the southern boreal zone, on average by about 200 degree days, between 1961 and 2012 (Fig. 2). Most of the observed increase in temperature and growing degree days has occurred since 1990 (see Fig. 2).

The number of species of conservation concern decreased from the period 1974–1989 to 2006–2010 (Table 2). However, there was a large variation in relation to the species distribution pattern, with northern species decreasing and southern species and species distributed over the whole country increasing in numbers. The decreasing trend for the numbers of all species considered was similar in all zones (interaction between period and zone nonsignificant) but the trends varied between the zones in the different distribution pattern groups (interaction significant). The number of southern species increased proportionally the most in the northern boreal zone and that of northern species decreased most in the southern boreal zone.

**Table 2:** Mixed-effect models where total species richness and species richness of species groups classified by their distributional patterns are related to atlas period (df = 1) and vegetation zone (df = 2) with interactions between these variables. Survey effort and a spatial autocovariate (ACV) are included as fixed covariates, and the grid square is included as a random effect term (see text). Statistical significances are based on *F*-tests for response variables with normal error structure (all species and species distributed over the whole country) for which linear mixed-effects models were fitted, but on Wald's χ^2^-test for response variables with Poisson error structure (southern and northern species) for which generalized mixed-effects models were fitted. For the *F*-test, df = 6676.

Species group	Survey effort		Spatial autocovariate		Period		Zone		Period × Zone	
	*F*/*χ*^2^	*P*	*F*/*χ*^2^	*P*	*F*/*χ*^2^	*P*	*F*/*χ*^2^	*P*	*F*/*χ*^2^	*P*
All species	5752.36	<0.001	2635.14	<0.001	52.25	<0.001 (−)	79.80	<0.001 (+)	1.54	0.214
Southern species	1160.33	<0.001	1645.98	<0.001	172.02	<0.001 (+)	3.57	0.167 (−)	57.68	<0.001
Northern species	1748.73	<0.001	1095.46	<0.001	270.44	<0.001 (−)	270.44	<0.001 (+)	83.82	<0.001
Species distributed over whole country	4393.04	<0.001	2939.19	<0.001	11.32	<0.001 (+)	13.22	<0.001 (+)	9.78	<0.001

Period + = increase, − = decrease between 1974–1989 and 2006–2010; zone + = increase toward the north, − = decrease toward the north.

**Figure 3:**
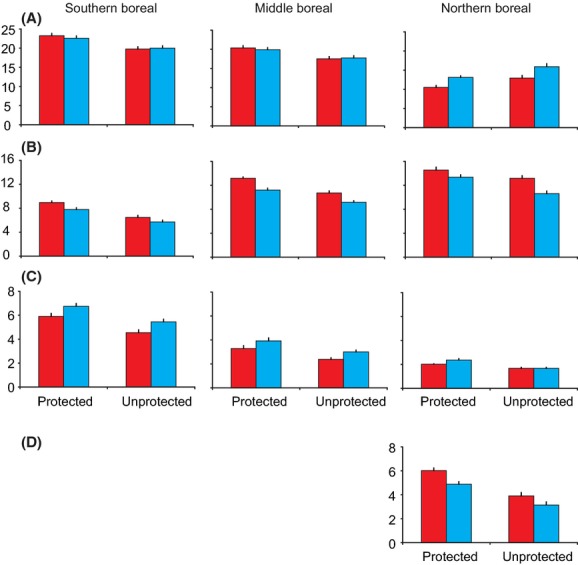
Mean (±SE) species numbers in southern boreal, middle boreal, and northern boreal zone in the different habitat types in protected and unprotected squares. A = forests, B = open mires, C = marshlands, D = Arctic mountain heaths. Red = 1974–1989, blue = 2006–2010. Note that these species numbers are based on original values affected by survey effort, which has been taken into account in the statistical analyses (Tables 2 and 3). For example, survey effort was higher in unprotected squares than in protected squares in northern boreal forests both in 1974–1989 (survey effort: protected squares = 2.64, unprotected squares = 3.06; *t* = 2.379, df = 98, *P* = 0.019) and in 2006–2010 (protected squares = 2.72, unprotected squares = 3.64; *t* = 5.622, df = 98, *P* < 0.001). In contrast, no statistical significance was observed in any of the comparisons in forests (*P* > 0.05 in all comparisons, *t*-test) between unprotected and protected squares in the southern and middle boreal zones in 1974–1989 and in 2006–2010.

When protected areas and habitat availability in the selected squares (Fig. 1) were included in the analysis, interesting patterns emerged. Here, species richness of forest species remained the same, species richness of marshland species increased and that of mire and Arctic mountain heath species decreased (Table 3, Fig. 3). Species richness of all species groups was higher in protected squares, and there was no interaction between the periods and protection status in forest, marshland, and Arctic mountain heath species. This suggests that the trends do not differ between protected and unprotected squares for these species. However, the slight interaction (*P* = 0.038) in mire species between period and protection is caused by species numbers declining more in unprotected than in protected squares.

**Table 3:** Mixed-effect models where species richness of the groups delineated by their primary habitat types are related to atlas period (df = 1), vegetation zone (df = 2), and protection status of the grid square (df = 1) with interactions between these variables. Survey effort is included as a fixed covariate, and the grid square is included a random effect term. Statistical significances are based on *F*-tests for response variables with normal error structure (forest, mire, and mountain heath species) for which linear mixed-effects models were fitted, but on Wald's χ^2^-test for response variables with Poisson error structure (marshland species) for which generalized mixed-effects models were fitted. For the *F*-test, df = 292–295 in forest and mire species, df = 97–98 in Arctic mountain species.

Species group	Survey effort		Period		Zone		Protection		Period × Zone		Period × Protection		Zone × Protection		Period × Zone × Protection	
	*F*/χ^2^	*P*	*F*/χ^2^	*P*	*F*/χ^2^	*P*	*F*/χ^2^	*P*	*F*/χ^2^	*P*	*F*/χ^2^	*P*	*F*/χ^2^	*P*	*F*/χ^2^	*P*
Forest species	928.08	<0.001	1.05	0.308 (+)	39.49	<0.001 (−)	27.43	<0.001 (+)	10.36	<0.001	0.39	0.535	9.18	<0.001	2.04	0.132
Mire sp.	62.19	<0.001	93.74	<0.001 (−)	307.85	<0.001 (+)	88.32	<0.001 (+)	0.292	0.747	4.33	0.038	1.00	0.369	4.11	0.017
Marshland sp.	64.74	<0.001	12.06	<0.001 (+)	200.08	<0.001 (−)	14.84	<0.001 (+)	0.854	0.652	0.16	0.685	0.42	0.812	1.23	0.541
Mountain sp.	34.55	<0.001	45.35	<0.001 (−)	–	–	61.81	<0.001 (+)	–	–	0.05	0.826	–	–	–	–

Period + = increase, − = decrease; zone + = increase toward the north, − = decrease toward the north; protection + = higher density in protected than in unprotected squares.

**Figure 4:**
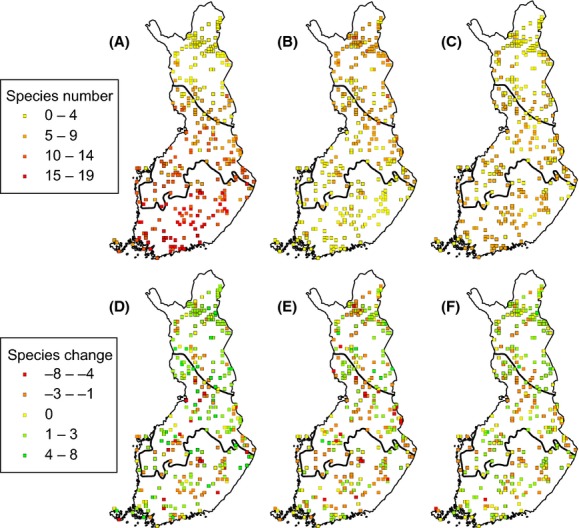
Species richness patterns of forest birds and their changes in the 150 protected and 150 unprotected 10-km grid cells. The upper panel shows species richness of forest birds in the period 1974–1989 grouped according to their distributional pattern: (A) southern, (B) northern, and (C) species occurring across the whole country. The lower panel shows change in species richness from the period 1974–1989 to the period 2006–2010 grouped according to their distributional pattern: (D) southern, (E) northern, and (F) species occurring in the whole country.

In forest birds there were significant interactions between period and zone as well as zone and protection status. This is due to the fact that species numbers increased in the northern boreal zone due to the expansion of southern species (Figs. 3 and 4), while species numbers remained the same or slightly declined in the southern and middle boreal zones (Fig. 3). The interaction in forest birds between zone and protection status is due to species numbers being higher in protected squares in the southern and middle boreal zones but in unprotected squares in the northern boreal zone (see Fig. 3). In the northern boreal zone, protected forest areas are concentrated in the northernmost part (Figs. 1 and S1), where most southern forest species do not occur (see Fig. 4). In addition, higher species numbers shown in Fig. 3 in unprotected than in protected forest squares in the northern boreal zone are affected by survey effort variation (taken into account as a cofactor in the model).

**Figure 5:**
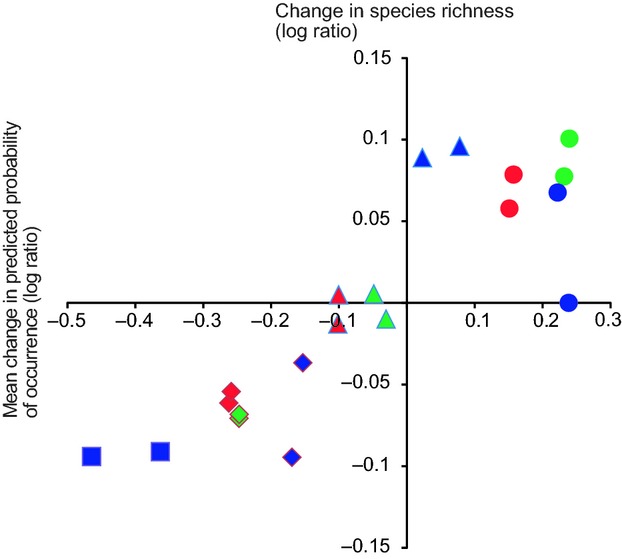
Change in species richness (from 1974–1989 to 2006–2010) in relation to mean change in predicted probability of occurrence of species (by 2051–2080) in the different boreal zones. Red = southern boreal, green = middle boreal, blue = northern boreal. Triangle = forest bird species, diamond = open mire species, circle = marshland species, square = Arctic mountain heath species. Each symbol category includes two signs, one for protected and one for unprotected areas. Both change in species richness and mean change in predicted probability of occurrence of species are based on logarithmic ratio (log ratio) of percentage changes.

The observed change in species richness in the different habitats in different boreal zones correlated highly positively with the mean long-term change in predicted probability of occurrence of species in corresponding habitats (*r* = 0.877, *P* < 0.001, *N* = 20, Fig. 5). For example, both observed species richness and predicted mean probability of occurrence of Arctic mountain heath and mire species have decreased and those of marshland species increased (see Fig. 5).

## Discussion

Previous studies have compared predictions of range shifts derived from bioclimatic envelope models (BEM) to the observed range changes (Araújo et al. 2005; Hijmans and Graham 2006; Eskildsen et al. 2013; Watling et al. 2013), but these studies have not considered how such changes are modified by the presence of protected areas. Our work shows that protected areas may significantly alleviate climate change effects on biodiversity. Moreover, the influence of climate warming is also reflected in species richness and abundance patterns in protected areas, but there are only a few studies which have made comparisons with predictions and real observational data (cf. Kharouba and Kerr 2010; Johnston et al. 2013). However, an essential finding in our results is that the landscapes with conservation areas have also maintained the higher level of species richness compared to unprotected areas during the recent period of climate warming.

Although we only included squares with focal habitat available in our analysis, the extent of the habitat in all habitat types, except in forests, was larger in protected than in unprotected squares. Species numbers are probably higher in landscapes with large protected areas due to the species-area effect of focal habitat (Rosenzweig 1995; Hanski et al. 2013) and because habitats in unprotected squares are often more fragmented (see Reino et al. 2013; Rybicki and Hanski 2013). Therefore, we suggest that this kind of area effect is likely to be one of the mechanisms causing higher species richness other than habitat quality in well-protected landscapes. Consequently, our findings provide support for the arguments that the extent of the protected area network has a central importance in preserving biodiversity in a warming climate (Hannah et al. 2007; Wiens et al. 2011; Thomas et al. 2012; Virkkala et al. 2013b). An additional effective factor is that habitats in protected areas are probably of higher quality than in unprotected areas. This is true, for example, for forest species, because many protected forests include stands of old-growth forest which are particularly important for many species of conservation concern (Virkkala and Rajasärkkä 2007).

An important issue for future conservation planning is how the present PA network will function and fulfill its goals in a changing climate (Hole et al. 2011; see also Geldmann et al. 2013). The PA network should enhance the survival of species in a changing climate, but so far there are scarce data to show this mitigation effect of protected area network on observed changes; see Bates et al. (2014), Johnston et al. (2013).

Despite the relatively short time span of the study period, the changes illustrated by our atlas data comparisons (between 1974–1989 and 2006–2010) show patterns that match well with the predictions made by Virkkala et al. (2013a,b). These include a decrease in species richness of mire and Arctic mountain heath species and an increase in marshland species. In forest species, ranges were predicted to decline most in southern and middle boreal areas and least in northern boreal areas (Virkkala et al. 2013a), and the observed changes between the periods 1974–1989 and 2006–2010 are largely congruent with these predictions. Consequently, our results suggest that the recent warming trend in the regional climate (cf. Heino et al. 2008; Tietäväinen et al. 2010; see Fig. 2) has already caused major abundance changes and range shifts in northern European bird species communities.

The matching trends between predicted and observed range shifts of boreal bird species suggest that predictive bioclimatic envelope models are useful in providing initial broad-scale impressions of the potential future changes in species ranges (Araújo et al. 2005; Heikkinen et al. 2006a, 2007; Araújo and Peterson 2012). It is also noteworthy that population changes of birds both in Europe (Gregory et al. 2009), the UK (Green et al. 2008), and Sweden (Jiguet et al. 2013) have correlated positively with the predictions of the BEMs. Nevertheless, there are clearly also cases where the model predictions have been contradicted by empirical evidence (see Araújo and Peterson 2012 and references therein). Thus, model validation plays a central role in separating cases and species with less successful model applications from the more robust ones. We advocate conducting validation tests in species–climate impact modeling whenever possible to increase the plausability of the projections (Araújo et al. 2005).

It seems that the geographic distribution of species is an important life-history characteristic affecting species richness changes in the different habitat types. In particular, southern species have expanded their ranges northwards, contributing to increased species richness, whereas northern species have retracted toward the north with a decreasing impact on species richness from 1974–1989 to 2006–2010. Most of the increasing marshland species were southern and all or almost all the decreasing Arctic mountain heath and open mire species, respectively, have a northern distribution pattern. By contrast, forest species consisted of both southern and northern species, showing increasing and decreasing trends in species richness, respectively. Similar changes have also occurred at a population level of bird species: the density of southern species increased and that of northern species decreased with density shifts northwards in protected areas of Finland between 1981–1999 and 2000–2009 (Virkkala and Rajasärkkä 2011a,b). Moreover, particularly the density of Arctic mountain and mire species have declined in protected areas (Virkkala and Rajasärkkä 2012) as also elsewhere in Fennoscandian mountains (Lehikoinen et al. 2013) and in Finland (Laaksonen and Lehikoinen 2013).

Some studies have suggested that protected areas are poorly located or cover too low proportion of land in terms of effectively preserving biodiversity in a changing climate in the future (e.g., Coetzee et al. 2009; Marini et al. 2009; D'Amen et al. 2011; Wiens et al. 2011; Velásquez-Tibatá et al. 2012). However, a BEM study focusing on PA networks on a European scale suggested that protected areas may retain climatic suitability of plant, mammal, bird, and reptile species by 2080 better than unprotected areas, although 58% of species may lose suitable climate in PAs (Araújo et al. 2011). This study also concluded that overall there may be more winners than losers in Finland and Sweden in both vertebrate and plant species in national protected areas in contrast with other European countries, where losers predominate over winners. Moreover, according to the analyses of Virkkala et al. (2013a), protected areas in Finland were not situated in suboptimal sites in relation to the predicted climate change, although in general climate is predicted to change more rapidly in the boreal forest biome than in any other biome in the world (Loarie et al. 2009).

Interestingly, Beale et al. (2013) found in Tanzanian savannah bird species that protected areas buffered the bird community against extinction, probably by limiting land degradation, and found no evidence that climate change to date was driving species away from protected areas. As with Tanzanian birds, the Finnish protected area mitigates the climate change effects on bird species of conservation concern and is predicted to preserve a high proportion of occurrences of these species in the future with the largest gaps, however, in forests in the southern and middle boreal zones (Virkkala et al. 2013b). In the UK, waterbird and seabird populations are predicted to decline considerably by 2080, but nevertheless protected areas remain highly important for the future conservation of these bird populations in a changing climate (Johnston et al. 2013).

In conclusion, it seems that the patterns of spatial optimality in the location of protected areas to preserving biodiversity in relation to climate change vary considerably between countries and biomes. In our study in boreal regions, we have shown that landscapes with significant amounts of protected areas alleviate the negative effects of climate warming on biodiversity. Such negative impacts are apparently more pronounced in unprotected areas due to their lower richness of species of conservation concern. Thus, instead of replacing protected areas (Fuller et al. 2010), the extent of protected areas should preferably be increased to better preserve biodiversity in the changing climate (Hannah et al. 2007). Future studies should concentrate on defining the major gaps in protected area networks in various habitats and biomes.

## Supporting Information

Appendix S1 Detailed presentation of CORINE land cover and distribution of protected areas.

Table S1 Studied species of conservation concern in different classifications.

Figure S1 Protected area network in Finland.
