# Supplementary material for: Protected areas alleviate climate change effects on northern bird species of conservation concern
Source: Ecol Evol. 2014 Jul 3;4(15):2991–3003. doi: 10.1002/ece3.1162 (PMC4161173; doi:10.1002/ece3.1162)
Supplement: Appendix S1 — Detailed presentation of CORINE land cover and distribution of protected areas. [file ece30004-2991-sd1.docx]

Supplementary Appendix S1. Detailed presentation of CORINE land cover and distribution of protected areas.

CORINE land cover

The classification of land cover into CORINE classes (in Finland) is based on the automated interpretation of Landsat ETM satellite images with a resolution of 30 m and data integration with existing digital maps (Härmä *et al.*, 2004). The proportion of land cover types was calculated as for each 10 x 10 km square and within each of the conservation area included in the study from a digital CORINE database using ArcView Spatial Analyst (Version 3.2, ESRI, Redland, CA, USA).

CORINE Land Cover 2006 was used to calculate the cover of the four main habitat types included in the study. Different coniferous, deciduous and mixed forest classes were combined as a single forest class, except mountain birch woodlands above the coniferous forest boundary in the Arctic mountains in the northern boreal zone. Open mires (i.e. treeless peatlands) were regarded as mire class. Marshland habitats (excluding mires) mostly occur along sea and lake shores and along rivers and in estuaries. The marshlands are largely dominated by the common reed *Phragmites australis* but also wet shoreline meadows are typical. Open, treeless Arctic mountain areas situated in the northern boreal zone were included as Arctic mountain heaths. The total land area of forests was 184,854 km^2^ (60.7% of total land area of 304,474 km^2^ in Finland), open mires 35,761 km^2^ (11.7%), marshlands 963 km^2^ (0.3%) and mountain heaths 7,368 km^2^ (2.4%).

Distribution of protected areas

We included here protected areas (national parks, strict nature reserves, mire conservation areas, bird wetland areas, old-growth forest areas, herb-rich forest areas, other protected areas on state-owned or privately owned land and wilderness areas), and areas included in officially accepted conservation programmes of specific habitats (old-growth forests, herb-rich forests, mires and bird wetland areas). All Natura 2000 areas, which are implemented by the Finnish nature conservation act (e.g. logging is not allowed) were also included in the protected habitat.

There are large (over 1000 km^2^ in size) national parks, wilderness areas and other protected areas in northernmost Finland. According to the CORINE Land Cover classification and digitised protected areas, 2.3% of forests in the southern boreal zone, 3.7% in the middle boreal zone and 23.4% in the northern boreal zone were included in protected areas. Corresponding figures of the proportion protected were 19.0%, 19.4% and 35.2% for open mires, and 26.7%, 24.2% and 12.1% for marshlands, respectively. In total, 88.9% of Arctic mountain heaths were situated in protected areas.

Selection of the focal grid squares

The 150 protected squares for forests included 33.0%, 39.5% and 26.5% of all protected forests in the southern, middle and northern boreal zones, respectively. The corresponding figures for protected mires were 56.9%, 44.1% and 27.1% and for marshlands 55.7%, 72.6% and 76.6% of their protected habitats in each zone. In Arctic mountain heaths, 51.3% of the protected habitats were situated in the protected squares.
