# Supplementary material for: Protected areas alleviate climate change effects on northern bird species of conservation concern
Source: Ecol Evol. 2014 Jul 3;4(15):2991–3003. doi: 10.1002/ece3.1162 (PMC4161173; doi:10.1002/ece3.1162)
Supplement: Table S1 — Studied species of conservation concern in different classifications. [file ece30004-2991-sd2.doc]

Table S1. Studied species of conservation concern in different classifications. DIR = EU BIRDS Directive species (Annex I), SPEC = species of European conservation concern (unfavourable conservation status: SPEC1–SPEC3, BirdLife International 2004a), IBA = species of Arctic or boreal biome (Heath & Evans 2000), EU = threatened species in European Union (unfavourable conservation status, BirdLife International 2004b), RES = species of special responsibility in Finland (Rassi et al. 2001), RED = red-listed species in Finland in 2010 (near-threatened and threatened species, Rassi et al. 2010), PREF = species preferring boreal old-growth or mature (coniferous or deciduous) forests (Väisänen et al. 1998, Virkkala et al. 1994, Virkkala & Rajasärkkä 2007). S = southern species, N = northern species, W = species distributed over the whole country. The data for three species (the Eurasian pygmy owl *Glaucidium passerinum*, the white-backed woodpecker *Dendrocopos leucotos* and the Arctic redpoll *Carduelis hornemanni*) included in Virkkala (2013a, 2013b) were not equally comparable between 1974–89 and 2006–10, and thus not included in the present analysis. For the pygmy owl, suitable nest boxes for the species have been provided in large amounts since the 1980s and due to this the number of observations has thus greatly increased in Finland (Saurola 2008, Valkama 2011). For the white-backed woodpecker, a detailed conservation and monitoring programme with intensive searching for the species started from 1990 onwards (Virkkala et al. 1993, Lehikoinen et al. 2011). The Arctic redpoll has been searched for particularly during the past 20 years with increased knowledge of its identification from the common redpoll *Carduelis flammea*, see Valkama (2011).

____________________________________________________________________________________________________________________

Species group DIR SPEC IBA EU RES RED PREF

____________________________________________________________________________________________________________________

Species of forests (N = 44)

Hazel grouse *Tetrastes bonasia* (S) + – – + – – –

Black grouse *Tetrao tetrix* (S) + + – + + + –

Western capercaillie *T. urogallus* (W) + – – + + + +

Black stork *Ciconia nigra* (S) + + – + – – –

Black kite *Milvus migrans* (S) + + – – – + +

European honey buzzard *Pernis apivorus* (S) + – – – – + –

Lesser spotted eagle *Aquila pomarina* (S) + + – + – – –

Golden eagle *A. chrysaetos* (N) + + – + – + +

Osprey *Pandion haliaetus* (W) + + – – – + –

Merlin *Falco columbarius* (N) + – – + – – –

Eurasian woodcock *Scolopax rusticola* (S) – + – + – – –

Eurasian eagle owl *Bubo bubo* (S) + + – – + + –

Northern hawk owl *Surnia ulula* (N) + – – – – – –

Ural owl *Strix uralensis* (S) + – + – – – –

Great grey owl *S. nebulosa* (N) + – + – – – –

Boreal owl *Aegolius funereus* (W) + – – – + + –

Eurasian nightjar *Caprimulgus europaeus* (S) + + – + – – –

Eurasian wryneck *Jynx torquilla* (S) – + – + – + –

Grey-headed woodpecker *Picus canus* (S) + + – + – – +

Black woodpecker *Dryocopus martius* (S) + – – – – – +

Lesser spotted woodpekcer *D. minor* (W) – – – – – – +

Three-toed woodpecker *Picoides tridactylus* (N) + + – + + – +

Wood lark *Lullula arborea* (S) + + – + – – –

Bohemian waxwing *Bombycilla garrulus* (N) – – + – – – –

Common redstart *Phoenicurus phoenicurus* (W) – + – + + – –

Mistle thrush *Turdus viscivorus* (S) – – – – – – +

Barred warbler *Sylvia nisoria* (S) + – – – – + –

Greenish warbler *Phylloscopus trochiloides* (S) – – – – – – +

Arctic warbler *Ph. borealis* (N) – – + – – + –

Wood warbler *Ph. sibilatrix* (S) – + – + – + –

Red-breasted flycatcher *Ficedula parva* (S) + – – – – – +

Collared flycatcher *F. albicollis* (S) + – – – – – –

Willow tit *Parus. montanus* (W) – – – + – – –

Siberian tit *P. cinctus* (N) – – + – – – +

Crested tit *P. cristatus* (S) – + – – – – –

Eurasian treecreeper *Certhia familiaris* (S) – – – – – – +

Eurasian golden oriole *Oriolus oriolus* (S) – – – + – + –

Siberian jay *Perisoreus infaustus* (N) – + + + + + +

Brambling *Fringilla montifringilla* (N) – – + – – – –

Two-barred crossbill *Loxia leucoptera* (N) – – + – – – –

Parrot crossbill *L. pytyopsittacus* (W) – – + – + – –

Common rosefinch *Carpodacus erythrinus* (S) – – – – – + –

Pine grospeak *Pinicola enucleator* (N) – – + – + – +

Rustic bunting *Emberiza rustica* (N) – – + + – + –

Species of mires ( N = 21)

Willow grouse *Lagopus lagopus* (N) – – – + – + –

Hen harrier *C. cyaneus* (N) + + – + – + –

Common crane *Grus grus* (W) + – – + – – –

Eurasian golden plover *Pluvialis apricaria* (N) + – – + – – –

Dunlin *C. alpina* (N) + + – + – + –

Broad-billed sandpiper *Limicola falcinellus* (N) – + – + + – –

Ruff *Philomachus pugnax* (N) + + – + – + –

Jack snipe *Lymnocryptes minimus* (N) – + + + + – –

Bar-tailed godwit *Limosa lapponica* (N) + – + + – – –

Whimbrel *Numenius phaeopus* (N) – – – + + – –

Spotted redshank *Tringa erythropus* (N) – + + + + – –

Common greenshank *T. nebularia* (N) – – + – + – –

Wood sandpiper *T. glareola* (N) + + – + + – –

Red-necked phalarobe *Phalaropus lobatus* (N) + – + – – + –

Short-eared owl *Asio flammeus* (N) + + – + – – –

Meadow pipit *Anthus pratensis* (N) – – – + – + –

Red-throated pipit *A. cervinus* (N) – – + + – + –

Yellow wagtail *Motacilla flava* (N) – – – + – + –

Great grey shrike *Lanius excubitor* (N) – + – + – – –

Lapland longspur *Calcarius lapponicus* (N) – – + – – – –

Little bunting *E. pusilla* (N) – – – + + – –

Species of marshlands (N = 15)

Great bittern *Botaurus stellaris* (S) + + – + – – –

Western marsh harrier *Circus aeruginosus* (S) + – – – – – –

Montagu's harrier *C. pygargus* (S) + – – – – + –

Spotted crake *Porzana porzana* (S) – – – – – + –

Little crake *P. parva* (S) – – – – – + –

Common snipe *Gallinago gallinago* (W) – + – + – – –

Great snipe *G. media* (S) + + – + – + –

Black-tailed godwit *Limosa limosa* (S) – + – + – + –

Common redshank *Tringa totanus* (W) – + – + – + –

Savi's warbler *Locustella luscinioides* (S) – – – – – + –

Great reed warbler *Acrocephalus arundinaceus* (S) – – – + – + –

Bearded parrotbill *Panurus biarmicus* (S) – – – – – + –

Penduline tit *Remiz pendulinus* (S) – – – – – + –

Yellow-breasted bunting *E. aureola* (S) – + – – – + –

Reed bunting *E. schoeniclus* (W) – – – + – – –

Species of Arctic mountain habitats (N = 10)

Rock ptarmigan *L. muta* (N*)* – – – + – – –

Rough-legged buzzard *Buteo lagopus* (N) – – + – – – –

Common ringed plover *Charadrius hiaticula* (N) – – – – – + –

Eurasian dotterel *C. morinellus* (N) + – – + – + –

Temminck's stint *Calidris temminckii* (N) – – + + – + –

Long-tailed skua *Stercorarius longicaudus* (N) – – + – – – –

Snowy owl *Bubo scandiacus* (N) + + + + – + –

Horned lark *Eremophila alpestris* (N) – – – + – + –

Bluethroat *Luscinia svecica* (N) + – – – – + –

Snow bunting *Plectrophenax nivalis* (N) – – + + – + –

___________________________________________________________________________________________________________________
