# Supplementary figures and images for: Protected areas alleviate climate change effects on northern bird species of conservation concern
Source: Ecol Evol. 2014 Jul 3;4(15):2991–3003. doi: 10.1002/ece3.1162 (PMC4161173; doi:10.1002/ece3.1162)

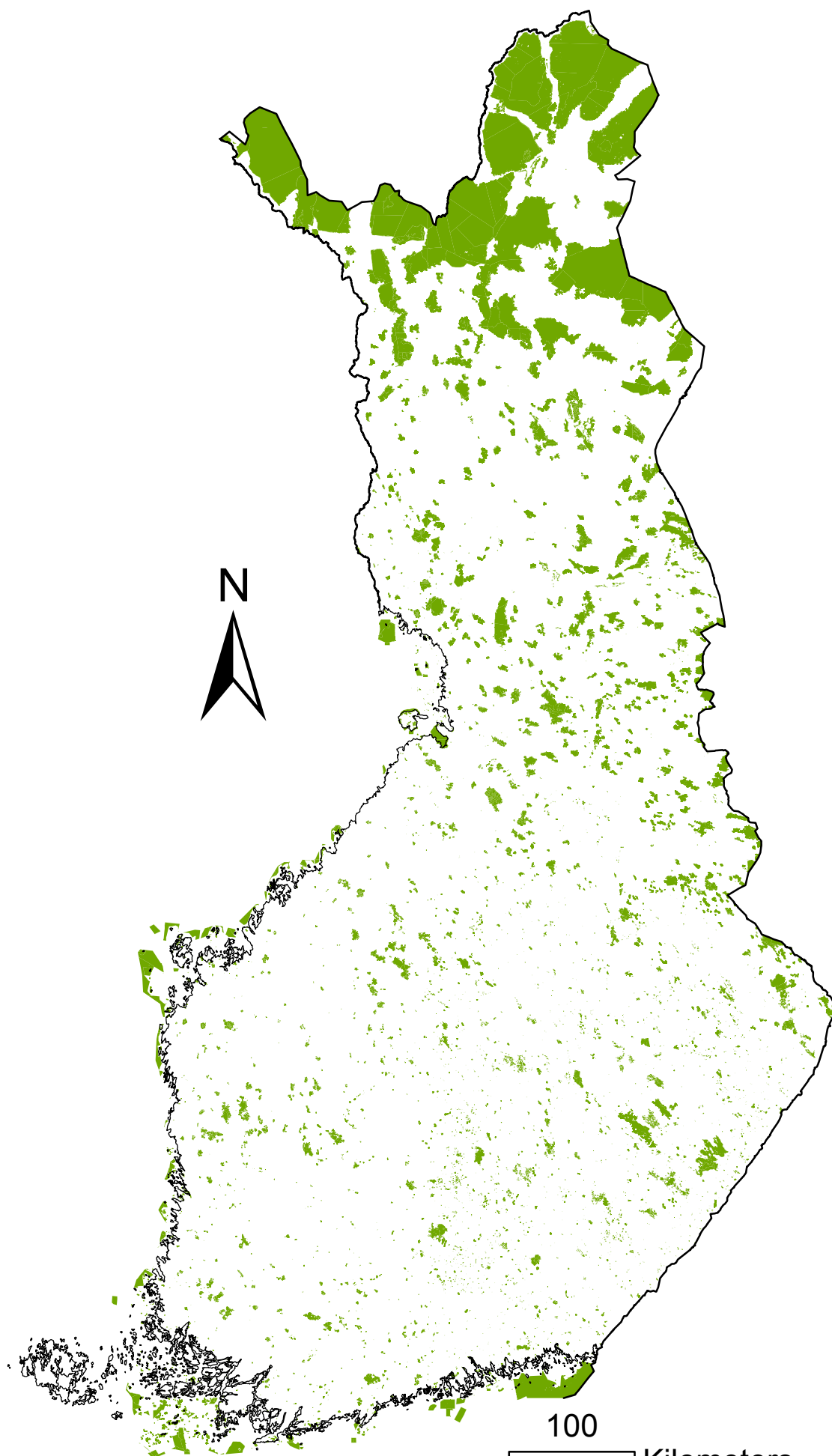

100  
Kilometers

Supplement: Figure S1 — Protected area network in Finland. [file ece30004-2991-sd3.pdf]
